# Milestone Age Affects the Role of Health and Emotions in Life Satisfaction: A Preliminary Inquiry

**DOI:** 10.1371/journal.pone.0133254

**Published:** 2015-08-05

**Authors:** Talya Miron-Shatz, Rajesh Bhargave, Glen M. Doniger

**Affiliations:** 1 Center for Medical Decision Making, Ono Academic College, Kiryat Ono, Israel; 2 Department of Marketing, University of Texas at San Antonio, San Antonio, Texas, United States of America; The Lieber Institute for Brain Development, UNITED STATES

## Abstract

Jill turns 40. Should this change how she evaluates her life, and would a similar change occur when she turns 41? Milestone age (e.g., 30, 40, 50)—a naturally occurring feature in personal timelines—has received much attention is popular culture, but little attention in academic inquiry. This study examines whether milestone birthdays change the way people evaluate their life. We show that life outlook is impacted by this temporal landmark, which appears to punctuate people’s mental maps of their life cycle. At these milestone junctures, people take stock of where they stand and have a more evaluative perspective towards their lives when making life satisfaction judgments. Correspondingly, they place less emphasis on daily emotional experiences. We find that milestone agers (vs. other individuals) place greater weight on health satisfaction and BMI and lesser weight on daily positive emotions in their overall life satisfaction judgments, whereas negative emotions remain influential.

## Introduction

Research has shown that various meaningful life events (e.g., marriage, graduation, etc.) impact life satisfaction [1], people’s general emotional states [2], and decisions that they make at specific points in life (e.g., career decisiveness while in college [3]). Yet, research has neglected milestone age (ages ending in 0; e.g., 40, 50, etc.), which occurs automatically and periodically in life. Milestone ages figure prominently in everyday conversations about age and life transitions. Hallmark promotes milestone birthday cards (e.g., “Forty and fabulous”) and certain self-improvement book titles draw attention to milestone ages (e.g., “Sex after Fifty…”. [4]; “…Flourishing after 60” [5]). This suggests that a temporal landmark can change people’s outlook and lead to an evaluative mode of thinking about one's life. We investigate this dynamic, studying how milestone ages influence how people construct life satisfaction judgments.

Milestone ages may be important landmarks in people’s “mental map of the life cycle”—their conception of life’s stages and transitions [6]. In particular, milestone ages punctuate mental maps, signifying a different life stage, due to a change in the first digit of one's age. Indeed, an approaching milestone age has recently been shown to drive people toward a search of meaningfulness [7]. Milestone ages are especially likely to signal shifts in life stage because people commonly treat round numbers, particularly multiples of 10, as reference points when perceiving stimulus categories [8–10]. Age is likely to be more salient to individuals in a milestone year, as is the notion of aging.

Phillips and Smith [11] defined milestone ages as symbolic events, during which one is more likely to engage in "stocktaking‴ (p. 81): the act of measuring where one stands in life. They found that in 1969–1985, suicide rates were higher for individuals at milestone ages. They attributed this result to enhanced attention to longstanding goals and future prospects, as well as the sense of hopelessness that such stocktaking may evoke. The spike in suicide rates was not limited to the days near a milestone birthday, but continued throughout the ensuing months, suggesting that the milestone age effect is caused by a prolonged shift in perspective.

"Stocktaking” [11] is related to the evaluative perspective in research on subjective well-being. According to this past work, when people assess their life, they rely on two perspectives [12–14]: (1) an emotional-experiential perspective encompasses the individual’s day-to-day positive and negative emotions; (2) an evaluative perspective encompasses thoughts about life currently and over time. We suggest that an emphasis on an evaluative (vs. emotional-experiential) perspective when constructing life satisfaction judgments will be greater for people at milestone ages (vs. other ages).

Individuals may consider various life domains when taking stock of their lives. We focus on health, because health-related variables strongly determine subjective age [15], and the incidence of health problems increases with age [16]. Further, health reflects past life choices and constrains future abilities. We suggest that if health is more salient during a milestone year because of a more evaluative perspective toward life satisfaction, daily emotion may contribute relatively less to life satisfaction judgments. We expect to find this effect as an indication to an overall shift to an evaluative (vs. emotional-experiential) perspective during milestone years.

This is the first work (to our knowledge) to examine life satisfaction and daily affect of milestone agers. In this pilot inquiry, we examine whether milestone agers (vs. others) weigh health satisfaction more in judging life satisfaction. To support the robustness of this predicted effect, we also evaluate whether milestone agers place greater weight on an objective health indicator–BMI—in constructing life satisfaction judgments, and if daily emotions are weighted less in life satisfaction judgments at milestone age. We explore the weighting of positive and negative daily emotional experiences separately, because negative emotions have been shown to be more impactful than positive emotions in general [17], and specifically, in well-being evaluations [18–20].

## Materials and Methods

### Participants

This is a secondary analysis of data from a Day Reconstruction Method (DRM) protocol [21]. Participants were 810 women from Columbus, Ohio. A survey company recruited them using random-digit dialing in the spring of 2005. Chronological ages ranged from 19 to 68 (*M* = 42.3,*SD* = 10.94). As age was unavailable for 10 participants, 800 women were included in the analyses. For additional details about the sample see Krueger et al. [22]. Participants provided written informed consent, and this study was approved by the institutional review board of Princeton University.

### Materials and Procedure

Participants first reported their life satisfaction and then completed a protocol designed to capture their daily experienced emotions. All participants followed the DRM [21], reconstructing the episodes of the previous day, from when they woke up until they went to sleep. For each episode, they specified what they did and the extent to which they experienced various feelings. Participants were then asked to report their feelings about different domains of life and respond to demographic measures.

### Measures

Participants responded to a question about general well-being, “Taking all things together, how satisfied are you with your life as a whole these days?” on a scale from 1 (“not at all satisfied) to 4 (“very satisfied”). They then rated the extent to which they felt positive emotions (happy, friendly, calm, competent, and interested; α = 0.91) and negative emotions (angry, tense, depressed, tired, and impatient; α = 0.84) during each episode on a scale from 0 (“not at all”) to 6 (“very much”). For positive and negative emotions separately, we summed these ratings to calculate average positive and average negative affect experienced across all episodes. These averages were weighted by the duration of the episodes, based on an existing methodology [21]. Participants later reported their health satisfaction; they responded to “How satisfied are you with your health these days?” on a scale from 1 (“not at all satisfied) to 4 (“very satisfied”). Participants reported their weight and height, with which we computed body mass index (BMI).

Participants reported their birth year, which was used to calculate their age at the time of the study, and their milestone status. Participants that reported a birth year that occurred 20, 30, 40, 50 or 60 years prior to the year when the survey took place were considered of milestone age. The data were collected in June, and as such, our operationalization of milestone age status comprises respondents that had a milestone birthday within the previous six months (January–June) or were about to have a milestone birthday in the next six months (July–December).

## Results

Milestone agers comprised 8.25% of participants, with equivalent life satisfaction ratings (*M* = 3.04, *SD* = 0.81) to others (i.e., non-milestone agers) (*M* = 3.01, *SD* = 0.72). Sub-groups of others by age digit (e.g., 1-ending, 2-ending, etc.) comprised between 9.13% (2-ending) and 11.50% (9-ending) of participants. Average chronological age did not differ between milestone-agers (*M* = 41.06) and others (*M* = 42.41; *t*(798) = 0.96, *p* = .34). We control for chronological age in the analyses, though results were similar without this control.

### Milestone age, health satisfaction and life satisfaction

**Fig 1:**
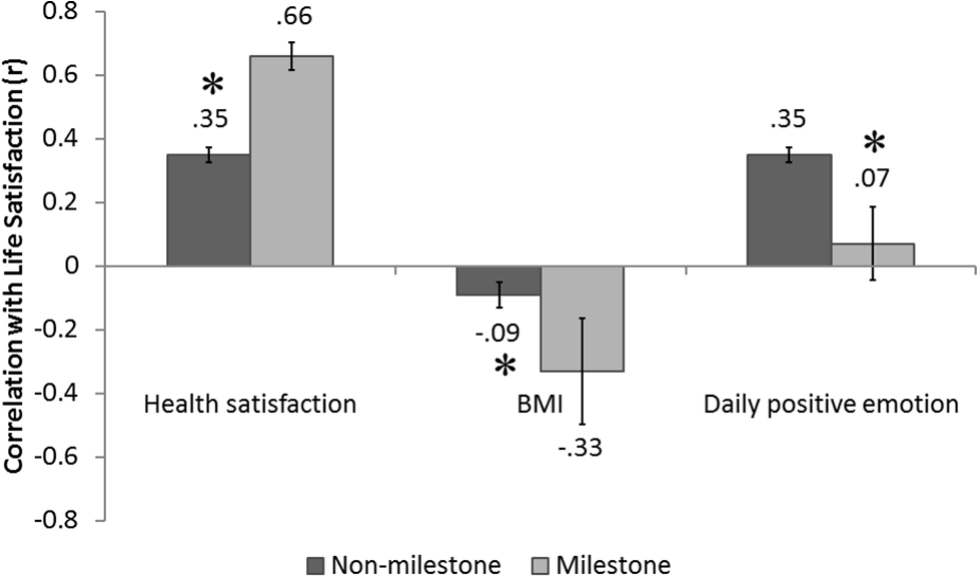
Correlates of life satisfaction. Relationships between life satisfaction and (1) health satisfaction (2) body mass index (BMI), and (3) daily positive emotions. The dark grey bar depicts correlation coefficients for non-milestone age participants, and the light grey bar depicts correlation coefficients for milestone age participants. Error bars represent standard error of the correlation coefficient. The asterisks denote significant (*p* < .05) differences between milestone age and non-milestone age participants (Fisher’s *Z* test).

We examined the relationship between health satisfaction and life satisfaction by milestone age status. Health satisfaction ratings were similar for milestone agers (*M* = 2.70, *SD* = 0.74) and non-milestone agers (*M* = 2.76, *SD* = 0.75). When examining the entire sample, life satisfaction had a sizeable relationship with health satisfaction (*r*(799) = 0.38,*p* < .001). This positive relationship was larger for milestone agers (*r*(66) = 0.66) than for others (*r*(733) = 0.35; both *p* < .001; Fisher’s *Z* = 3.26, *p* = .001) (Fig 1).

Life satisfaction was subjected to an ANOVA with independent factors of health satisfaction, milestone status, and their interaction, controlling for chronological age. There was a significant main effect of health satisfaction (*F*(1,794) = 81.90, *p* < .001) and a significant main effect of milestone status (*F*(1,794) = 8.92, *p* < .001). Most importantly, we also found a significant health satisfaction X milestone-status interaction (*F*(1,794) = 10.71, *p* = .001), consistent with our assertions. Chronological age was not associated with health satisfaction (*r*(799) = -0.01). Moreover, we analyzed the relationship between health satisfaction and life satisfaction over the chronological age range. Life satisfaction was subjected to an ANOVA with only the following independent factors: health satisfaction, chronological age, and their interaction. This analysis showed no interaction (*F*(1,795)<1).

### Milestone age and objective indicators of health

To establish that the milestone effect in the evaluation of life satisfaction extends beyond subjective predictors, we examined the milestone age effect on BMI, an objective measure of health (e.g., [23]). The relationship between BMI and life satisfaction was larger for milestone agers (*r*(66) = -0.45) than for others (*r*(730) = -0.09; both *p* < .05 Fisher’s *Z* = -3.00, *p* = .003) (Fig 1). We subjected life satisfaction to an ANOVA with independent factors of BMI, milestone status, and their interaction, controlling for chronological age. There was a significant main effect of BMI (*F*(1,791) = 22.86, *p* < .001) and a significant BMI X milestone-status interaction (*F*(1,791) = 7.17, *p* < .01), consistent with our predictions. Greater chronological age showed an association with higher BMI (*r*(799) = 0 .11, *p* < .01), but when modeling life satisfaction as a function of BMI, chronological age, and their interaction, there was no BMI X chronological age interaction (*F*(1,792)<1).

We tested whether milestone agers heavily weighted both BMI and health satisfaction, or whether these effects were redundant, given the significant relationship between BMI and health satisfaction (*r*(796) = -0.32, *p* < .001). We subjected life satisfaction to an ANOVA with independent factors of BMI, health satisfaction, milestone status, and the two interactions with milestone status ((1) BMI X milestone status, and (2) health satisfaction X milestone status), controlling for chronological age. The health satisfaction X milestone status interaction held (*F*(1,789) = 4.57, *p* = .03), but the BMI X milestone status interaction only trended toward significance (*F*(1,789) = 3.56, *p* = .06). This analysis suggests that milestone agers exhibited a general tendency to weight health more in constructing life satisfaction, and subjective health satisfaction may have been more relevant to life satisfaction than objective BMI.

### Milestone age and the weighting of daily affect in life satisfaction judgments

We next tested whether milestone agers would underweight emotional experiences relative to others. Duration-weighted positive affect was similar for milestone agers (*M* = 4.19, *SD* = 0.95) and non-milestone agers (*M* = 3.99, *SD* = 1.05). Milestone agers exhibited little relationship between positive emotions and life satisfaction (*r*(66) = 0.07, *p*>.5), but more positive emotion was associated with greater life satisfaction for non-milestone agers (*r*(734) = 0.35, *p <* .001; Fisher’s *Z* = -2.25, *p* = .025) (Fig 1).

We subjected life satisfaction to an ANOVA with independent factors of milestone status, positive emotions, and health satisfaction, along with the positive emotions X milestone status, and the health satisfaction X milestone status interactions, controlling for age. Both the positive emotions X milestone-status (*F*(1,792) = 7.76, *p* < .01) and the health satisfaction X milestone-status (*F*(1,792) = 15.98, *p* < .001) interactions were significant in this model that considered their joint effects (Fig 1).

Duration-weighted negative affect was similar for milestone agers (*M* = 1.06, *SD* = 0.93) and non-milestone agers (*M* = 1.16, *SD* = 0.97). We found that the relationship between life satisfaction and negative emotions did not differ by milestone status (*r*_*ms*_(66) = -0.4 vs. *r*_*non-ms*_(734) = -0.34; Fisher’s *Z* = -.53, *p* = .596). We subjected life satisfaction to an ANOVA with independent factors of milestone status, negative emotions, and health satisfaction, along with the negative emotions X milestone status, and the health satisfaction X milestone status interactions, controlling for age. The health satisfaction X milestone-status interaction held (*F*(1,792) = 9.05, *p* < .01), but there was no negative emotions X milestone-status interaction (*F*(1,792) = 0.28, *p*>.5) (see Fig 1).

We then sought to better understand whether these differences in weighting of predictors of life satisfaction are unique to milestone agers or would also be found in smaller sub-samples classified by the ones digit in their ages. Specifically, we computed the relationship between life satisfaction and (1) health satisfaction, (2) BMI, and (3) daily positive emotions separately for the ten groups (i.e., 0-ending milestone ages, 1-ending ages, 2-ending ages, etc.). These correlations are presented in Table 1.

**Table 1:** Relationship between life satisfaction and (1) health satisfaction, (2) BMI, and (3) daily positive emotions, separately for groups classified by the ones (i.e., last) digit in their age.

Age group	r (health sat with life sat)	r (BMI with life sat)	r (positive emotions with life sat)
ending-0 (milestone)	**0.66**	**-0.45**	0.07
ending-1	0.13	-0.15	**0.4**
ending-2	**0.38**	-0.08	**0.35**
ending-3	**0.29**	-0.2	**0.38**
ending-4	**0.57**	-0.14	**0.31**
ending-5	**0.25**	0.1	**0.46**
ending-6	**0.36**	-0.07	**0.26**
ending-7	**0.34**	0.02	**0.24**
ending-8	**0.47**	-0.05	**0.43**
ending-9	**0.33**	-0.18	**0.36**

Bold values are significant.

Health satisfaction showed a significant positive correlation with life satisfaction for all but one of these 10 groups. Still, this relationship was highest among milestone agers. BMI was only significantly correlated with life satisfaction for milestone agers, and not for others. Finally, positive emotions were significantly correlated with life satisfaction for all age groups but milestone agers. Thus, when examined across the sample, the milestone age effect did not carry over to adjacent years (i.e., 1-ending, 9-ending ages), and the pattern was different in other years, thereby suggesting that the construction of life satisfaction judgments at milestone ages is distinct.

## Discussion

This research focuses on milestone age, a naturally occurring juncture in people’s mental maps of their life cycles, and describes its effect on the construction of life satisfaction judgments. We found that milestone agers (compared with non-milestone agers) weighted health satisfaction more, and positive emotions less, in life satisfaction. Both of these changes in weighting were significant in analyses controlling for their joint effects. These results are consistent with our assertions that people have a more evaluative (vs. emotional-experiential) perspective when assessing their lives in milestone years. We also found that although the influence of positive emotion in life satisfaction judgments was reduced for milestone agers, the influence of daily negative emotions remained similar to non-milestone agers. This finding is consistent with past research that has shown that negative (relative to positive) emotions are more impactful on people’s judgments [17], including those concerning life assessment [18–20].

Interestingly, Wirtz and colleagues [24] suggest that European Americans are more prone to basing life satisfaction judgments on positive events and emotions, whereas their Asian American counterparts focus more on negative events and emotions. Future work might examine cross-cultural differences in the weight milestone agers place on health in their life satisfaction judgments. Other work by Siedlecki and colleagues [25] shows that perceived support and a sense of embeddedness contribute to life satisfaction across ages. It might be fruitful to examine the weight of this variable in milestone years, potentially in conjunction with an examination of whether the participant's culture is predominantly individualistic or collectivist [26] (see also [27]).

The measures and analyses reported control for the impact of potential biases and confounding variables: it is unlikely we encountered a bias against reporting milestone age because participants reported their birth year, not their age. Moreover, chronological age did not behave comparably to milestone age status in our regression models. This likely further supports our findings and our interpretation of an association between milestone and a shift in one's evaluative perspective. As this shift is temporary, it follows that it is not one's actual age that affects judgment, but rather the focus on one's age (as elicited by a milestone). We also revealed that milestone agers were distinct in how they constructed life satisfaction by comparing milestone agers to other sub-samples of similar sizes (e.g., 1-ending ages, 2-ending ages, etc.). Finally, we found that milestone agers (vs. others) weighted both health satisfaction and BMI more in life satisfaction, although health satisfaction absorbed most of the effect of BMI when both measures were included as predictors. Nonetheless, our assertions are strengthened by similar results with both objective and subjective measures of health.

Our data, while collected in a relatively homogeneous sample, helps establish that a temporal landmark affected how participants judge life satisfaction. Temporal landmarks may differ across populations, as may the life domains associated with them, but this work is an important first step in revealing that there is such an association. The present study was limited to a small sample of milestone agers as the survey was not initially designed to examine milestone agers, and we did not actively recruit them. Although this bolsters the ecological validity of our preliminary findings, future studies should selectively target milestone agers to obtain a larger sample. Similarly, our research was carried out exclusively in women, but some work suggests that gender influences how people perceive symbolic events [28]. Future research should examine whether the obtained effects in women apply equally to men.

Additionally, future research may test for differences among specific milestone ages (e.g., 40 vs. 50) by focusing solely on these milestone ages. Researchers may also examine whether milestone agers make life choices consistent with enhanced salience of personal health, such as more frequent checkups and visits to doctors, increased purchases of health and lifestyle products, etc. The present findings have implications for public policymakers that may cater to milestone agers' pronounced emphasis on health. For instance, milestone agers might be a natural target group for services that emphasize health and mark one's life achievements, such as screening for particular age-related illnesses. In sum, milestone age may prompt not only a blowout birthday celebration, but also substantive changes in life outlook.

## Supporting Information

S1 File Dataset. (XLSX)
